# Transcriptional Regulation of N-Acetylglutamate Synthase

**DOI:** 10.1371/journal.pone.0029527

**Published:** 2012-02-27

**Authors:** Sandra Kirsch Heibel, Giselle Yvette Lopez, Maria Panglao, Sonal Sodha, Leonardo Mariño-Ramírez, Mendel Tuchman, Ljubica Caldovic

**Affiliations:** 1 Center for Genetic Medicine Research, Children's National Medical Center, Washington, D. C., United States of America; 2 Molecular and Cellular Biology Program, University of Maryland, College Park, Maryland, United States of America; 3 Department of Pathology, Duke University Medical Center, Durham, North Carolina, United States of America; 4 The George Washington University School of Medicine and Health Sciences, Washington, D. C., United States of America; 5 Johns Hopkins School of Medicine in Baltimore, Maryland, United States of America; 6 Computational Biology Branch, National Center for Biotechnology Information, National Library of Medicine, National Institutes of Health, Bethesda, Maryland, United States of America; Texas A&M University, United States of America

## Abstract

The urea cycle converts toxic ammonia to urea within the liver of mammals. At least 6 enzymes are required for ureagenesis, which correlates with dietary protein intake. The transcription of urea cycle genes is, at least in part, regulated by glucocorticoid and glucagon hormone signaling pathways. N-acetylglutamate synthase (NAGS) produces a unique cofactor, N-acetylglutamate (NAG), that is essential for the catalytic function of the first and rate-limiting enzyme of ureagenesis, carbamyl phosphate synthetase 1 (CPS1). However, despite the important role of NAGS in ammonia removal, little is known about the mechanisms of its regulation. We identified two regions of high conservation upstream of the translation start of the *NAGS* gene. Reporter assays confirmed that these regions represent promoter and enhancer and that the enhancer is tissue specific. Within the promoter, we identified multiple transcription start sites that differed between liver and small intestine. Several transcription factor binding motifs were conserved within the promoter and enhancer regions while a TATA-box motif was absent. DNA-protein pull-down assays and chromatin immunoprecipitation confirmed binding of Sp1 and CREB, but not C/EBP in the promoter and HNF-1 and NF-Y, but not SMAD3 or AP-2 in the enhancer. The functional importance of these motifs was demonstrated by decreased transcription of reporter constructs following mutagenesis of each motif. The presented data strongly suggest that Sp1, CREB, HNF-1, and NF-Y, that are known to be responsive to hormones and diet, regulate *NAGS* transcription. This provides molecular mechanism of regulation of ureagenesis in response to hormonal and dietary changes.

## Introduction

Ammonia, the toxic product of protein catabolism, is converted to urea by the urea cycle in the liver of mammals. Incorporation of two nitrogen atoms into urea is catalyzed by six enzymes: three of them mitochondrial, N-acetylglutamate synthase (NAGS; EC 2.3.1.1), carbamylphosphate synthetase 1 (CPS1; EC 6.4.3.16) and ornithine transcarbamylase (OTC; EC 2.1.3.3), and the other three cytosolic, argininosuccinate synthetase (ASS; EC 6.3.4.5), argininosuccinate lyase (ASL; EC 4.3.2.1) and arginase 1 (Arg1; EC 3.5.3.1).

NAGS catalyzes the formation of N-acetylglutamate (NAG), an essential allosteric activator of CPS1, in the mitochondrial matrix of hepatocytes and small intestine epithelial cells [1], [2]. Within hepatocytes, NAGS activity and NAG abundance are regulated by L-arginine, ammonia, and dietary protein intake [3], [4], [5] and therefore, the NAGS/NAG system may play a critical role in the regulation of ureagenesis in response to these factors [6]. While studies in the 1980s and 1990s identified the *cis*-acting motifs regulating transcription of the urea cycle enzymes CPS1 [7], [8], [9], [10], OTC [11], [12], [13], [14], ASS [15], [16], [17], ASL [18], [19], [20], and Arg1 [21], [22], the mammalian *NAGS* gene was not identified until 2002 [2] and we can now report for the first time on its transcriptional regulation.

Many studies have identified regulatory links between the urea cycle genes and glucocorticoids and glucagon [23], [24], [25], however the mechanism of regulation differs for each gene [24], [26], [27], [28], [29]. Transcription of *CPS1* is activated by TATA-binding protein (TBP) while its proximal and distal enhancers contain binding sites for glucocorticoids and cAMP responsive factors including CCAAT-enhancer bind protein (C/EBP), activator protein-1 (AP-1), glucocorticoid receptor (GR) and cAMP response element binding (CREB). Sites for binding tissue specific factors including hepatic nuclear factor 3 (HNF-3) are also present [25], [30], [31]. Tissue specific expression of the *OTC* gene is induced in the intestine and liver by HNF-4, which binds in the promoter [13], [14], [32] while binding of both HNF-4 and C/EBP to the enhancer, induces high expression levels in the liver [12], [13], [14], [25], [33]. *ASS* transcription is regulated by cooperative binding of multiple specificity protein 1 (Sp1) 16,34,35,36. *ASL* is regulated through Sp1 and the positive regulator, nuclear factor Y (NF-Y), which binds within the promoter of *ASL* to activate its transcription [18], [19], [20], [37]. Sp1 and nuclear factor 1 (NF-1)/CCAAT-binding transcription factor (CTF) activate *ARG1* transcription while two C/EBP factors and two unidentified proteins bind within an enhancer in intron 7 to confer glucocorticoid responsiveness [22].

Abundance of urea cycle enzymes correlates with dietary protein intake [3], [28]. Transcription of urea cycle genes is in part regulated by the glucocorticoid and glucagon signaling pathways [29], [38]. Therefore, we postulate that there exists a nitrogen sensing mechanism that is both responsive to amino acid(s) and hormone stimulation and that an understanding of the transcriptional regulation of *NAGS* could contribute to the understanding of such mechanism.

In this study, we identified two regulatory regions upstream of the NAGS translation start site that contain highly conserved protein-binding DNA motifs. We subsequently confirmed that these regions function as promoter and enhancer and that the enhancer is most effective in liver cells. Avidin-agarose protein-DNA pull-down assays have been used to confirm binding of Sp1 and CREB within the *NAGS* promoter and Hepatic Nuclear Factor 1 (HNF-1) and NF-Y within the enhancer regions. Chromatin immunoprecipitation (ChIP) and quantitative real-time PCR have been used to independently verify that Sp1 and CREB bind to the promoter region, and HNF-1 and NF-Y bind to the enhancer region. We also used 5′RACE analysis to identify multiple transcription start sites for *NAGS* that may be species and tissue specific. These findings provide new information on the regulation of the *NAGS* gene, and suggest possible mechanisms for coordinated regulation of the genes involved in ureagenesis.

## Materials and Methods

### Bioinformatic Analysis of the Upstream Regulatory Regions

#### Pair-wise Alignment Analysis

Identification of highly conserved regions was conducted by gathering 15 kilobases of genomic sequence 5′ of the *NAGS* translational start site and sequence of intron one in 7 mammalian species including: human (NM_153006.2), chimpanzee (XM_001152480.1), dog (XM_548066.2), cow (XM_618194.4), horse (XM_001917302.1), mouse (NM_145829.1) and rat (NM_001107053.1). The highly conserved regulatory regions of *CPS1* were identified by gathering 15 kilobases of genomic sequences 5′ of the translational start site from human (NM_001875), chimpanzee (XM_001146604), dog (XM_856862), mouse (NM_001080809), and rat (NM_017072). Genomic sequences were subject to pair-wise comparison using BLAST bl2seq tool [39]. Parameters included expect threshold of 10, match and mismatch scores of 1 and −2, respectively, gap existence and extension scores of 5 and 2 respectively, and maximum expected value E = 0.001. Regions of high conservation were identified as sequences with more than 80% identity that were at least 100 bp long and present in four or more species.

#### Cis-eLement OVERrepresentation (CLOVER) Analysis

The *Cis*-element OVERrepresentation (CLOVER) [40] program was used to predict the over-represented motifs within the highly conserved regulatory regions of *NAGS* and *CPS1*. CLOVER analysis of these conserved regions identified known protein binding DNA motifs in the TRANSFAC Pro database by calculating over-representation of these sequences compared to a background of ppr_build_33.fa generated from NCBI build 33 [41]. Matrices recognized by multiple transcription factors in the same family are represented by one family member unless otherwise noted. Genomic sequences of the highly conserved regions were aligned using CLUSTALW version 2.0.10 [42].

### Plasmid Constructs

The promoter and enhancer of *NAGS*, were amplified from human genomic DNA with primer pairs hPromXH and hEnhXH or hPromHXrev and hEnhHXrev (Table S1), respectively, to introduce *XhoI* and *HindIII* restriction enzyme sites and allow subcloning in forward and reverse orientation. Platinum Taq PCRx DNA Polymerase (Invitrogen) was used for amplification with the following conditions: initial denaturation at 95°C for 2 min., followed by 35 cycles of denaturation at 95°C for 30 sec., annealing at 57°C for 30 sec. and extension at 68°C for 1 min., and final extension at 68°C for 6 min. Promoter and enhancer PCR products were ligated with TOPO-TA sequencing vector (Invitrogen) according to manufacturer's instructions and referred to as TOPOProm, TOPOEnh, TOPOPromRev, and TOPOEnhRev, respectively. Mouse *Nags* (m*Nags*) promoter and enhancer were inserted into TOPO-TA vector following the same methods. Correct DNA sequences were confirmed using sequencing primers specified by Invitrogen.

TOPOProm, TOPOEnh, TOPOPromRev, TOPOEnhRev, pGL4.10 (Promega) basic vector containing firefly (*Photinus pyralis*) luciferase *luc2*, and pGL4.23 (Promega) vector containing a minimal TATA promoter with *luc2* were cut with *XhoI* (New England Biolabs) and *HindIII* (New England Biolabs). The vectors were treated with Antarctic Alkaline Phosphatase (AAP) (New England Biolabs) according to manufacturer's instructions, and the *NAGS* regions were ligated with the vectors to form the plasmids in Table 1. TOPOEnh was also amplified with primer pair hEnhBS (Table S1), to introduce *BamHI* and *SalI* restriction enzyme sites at the 5′ and 3′ ends of the enhancer, respectively. The amplified enhancer product and 4.10Prom were cut with *BamHI* (New England Biolabs) and *SalI* (New England Biolabs), the vector was treated with AAP, and the enhancer was ligated with the vector (Table 1). Plasmids containing mouse *NAGS* promoter and enhancer were generated using the same methods with the primer pairs listed in Table S1 and plasmids in Table 1. Correct sequences were confirmed using primers specified by Promega.

**Table 1:** Plasmids generated for luciferase reporter assays.

Name	Vector	Insert
4.10Prom	pGL4.10	hNAGS promoter
4.10Enh	pGL4.10	hNAGS enhancer
4.23Enh	pGL4.23	hNAGS enhancer
4.10PromEnh	4.10Prom	hNAGS enhancer
4.10PromRev	pGL4.10	hNAGS promoter reverse
4.23EnhRev	pGL4.23	hNAGS enhancer reverse
m4.10Prom	pGL4.10	mNAGS promoter
m4.10Enh	pGL4.10	mNAGS enhancer
m4.23Enh	pGL4.23	mNAGS enhancer
m4.10PromEnh	4.10Prom	mNAGS enhancer
4.10Sp1m	pGL4.10	hNAGS promoter with Sp1 mutations
4.10CREBm	pGL4.10	hNAGS promoter with CREB mutations
4.23HNF-1m	pGL4.23	hNAGS enhancer with HNF-1 mutations
4.23NF-Ym	pGL4.23	hNAGS enhancer with NF-Y mutations

Human or mouse promoter or enhancer were ligated with pGL4 vectors for use with luciferase reporter assays.

Point mutations in the binding sites for transcription factors Sp1, HNF-1 and NF-Y were selected based on functional analysis of Sp1 [43], [44], [45], HNF-1 [46], [47], and NF-Y [48], [49] binding in other genes. Mutations were engineered by Integrated DNA Technologies and provided in pIDTSMART-KAN vectors (IDT) (Table 2). Plasmids with mutant Sp1, HNF-1, and NFY were cut with *XhoI* and *HindIII*. Reporter plasmids pGL4.10, and pGL4.23 were cut with *XhoI* and *HindIII* and treated with AAP. Mutated inserts were ligated with vectors to form the plasmids 4.10Sp1m, 4.23HNF-1m, and 4.23NFYm (Table 1). Correct sequences were confirmed using primers specified by Promega.

**Table 2:** Mutations in Sp1 and CREB binding sites in the promoter, and HNF-1 and NF-Y in the enhancer of human *NAGS*.

Factor	Wild-type	Mutant
Sp1	5′-CCGCCCCCGCC-3′	5′-AAGAACAAGAA-3′
	5′-GGGGCGGGGG-3′	5′-GGTTCTTTGG-3′
	5′-CCCCGCCCCC-3′	5′-CCAAGAAACC-3′
	5′-CCCCGCCCCG-3′	5′-CCAAGAAACG-3′
CREB	5′-GGTTGTCGTCATGG-3′	5′-GGTCGACGTCATGG-3′
HNF-1	5′-TGGAGTTAATCATCTACTCTG-3′	5′-TGGAGTAAGTCTGCAACCAGG-3′
NF-Y	5′-GGCCCCATTGGCTGCCT-3′	5′-GGCCCCTCCAGCTG-3′

Point mutations in the CREB binding site, c.-7T>C and c.-5T>A (Table 2), were selected based on functional analysis of CREB binding [50], [51] in other genes and were engineered into the *NAGS* gene using QuickChange Lightening Site-Directed Mutagenesis Kit (Agilent) according to manufacturer's instructions. Primers hCREBm Fw and Rv (Table S1) amplified 50 ng of template plasmid 4.10Prom to create 4.10CREBm. The correct sequence was confirmed using primers specified by Promega.

The expression vectors encoding Sp1 or HNF-1 cDNA were under control of the cytomegalovirus promoter (Origene).

### Tissue culture

#### Cell culture and transfection

Human hepatoma cells (HepG2) (donated by Dr. Marshall Summar, Children's National Medical Center, Washington, DC) were cultured in complete media containing RPMI 1640 medium (Invitrogen) supplemented with 10% fetal bovine serum (FBS) (ATCC) and 5% Penicillin/Streptomycin (Invitrogen) under 5% CO_2_ at 37°C. Human alveolar basal epithelial cells (A549) (donated by Dr. Mary Rose, Children's National Medical Center, Washington, DC) were cultured in complete media containing Ham's F-12 medium (Invitrogen) supplemented with 10% FBS and 5% Penicillin/Streptomycin. Human colorectal adenocarcinoma cells (Caco-2) (ATCC) were cultured in Eagle's Minimum Essential Medium (Invitrogen) supplemented with 20% FBS. Cells were plated at a density of 5×10^5^ cells/well on 24-well culture plates 24 hours prior to transfection. The cells (90–95% confluent for HepG2 and A549, 80–85% confluent for Caco-2) were then transfected using Lipofectamine 2000 reagent (Invitrogen) and cultured in transfection media containing medium and serum only. A total of 0.25 ug of DNA was transfected with 0.225 ug of vector expressing *luc2* and 0.025 ug of pGL4.74 vector containing *Renilla reniformis* luciferase (*hRluc*) as an internal control (Promega). For co-transfections 0.225 ug of *luc2* vector was combined with either 0.25 ug of expression vector or empty vector pUC19 (Invitrogen), and 0.025 ug of *hRluc* control vector.

### Reporter assays

24 hours following transfection, cells were assayed for both firefly and *Renilla* luciferase activity using Dual-Luciferase Reporter Assay System (Promega) and Berthold Centro 960 luminometer (Berthold) according to the manufacturer's protocol. All reporter assay measurements were corrected for transfection efficiency by normalizing the firefly luciferase signal to the *Renilla* luciferase values. Expression level of each construct was determined relative to luciferase expression under control of the *NAGS* promoter in each cell line. All results are an average of three independent experiments that were each carried out in triplicate. Values were expressed as mean ± SEM and analyzed using Student's *t*-test.

### 5′ Rapid Amplification of cDNA Ends (RACE)

5′ RACE (Version 2.0; Invitrogen) was performed using RNA isolated from donated mouse livers by Trizol reagent (Invitrogen). RNA from mouse small intestine (Origene), human duodenum (Ambion), or human liver (Ambion) was commercially available. Products were synthesized with human or mouse *NAGS* specific primers complementary to sequence within Exon 1 (Table S2). All reactions began with 5 ug of total RNA and the RACE procedure was conducted according to manufacturer's instructions. Second strand synthesis was conducted using Ex Taq Polymerase (TaKaRa Bio Inc.) PCR products were subcloned into pCR 2.1-TOPO vector (Invitrogen) and RACE products were sequenced with primers specified by the manufacturer.

### Avidin-Agarose DNA-Protein Pull-Down Assay

#### Biotinylated DNA probes

**Figure 1:**
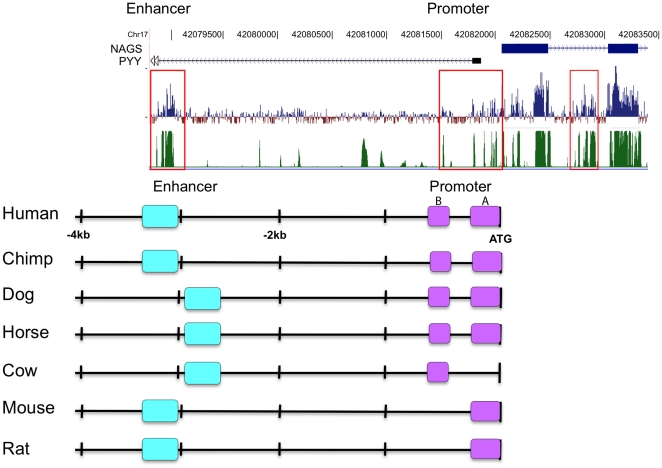
Regions upstream of the mammalian *NAGS* genes that are highly conserved. Conservation of mammalian *NAGS* DNA by phastCons (green) and phyloP (blue) algorithms is shown with the highly-conserved regions indicated in red boxes (A). Pair-wise blast analysis of mammalian non-coding regions of *NAGS* identified highly conserved sequences upstream of the translational start site termed the promoter (purple) and enhancer (cyan) (B).

Probes for Avidin-Agarose DNA-Protein Pull-Down Assays were generated by PCR amplification of genomic DNA isolated from donated mouse tails using Pure Gene DNA Purification Kit (Gentra). Probes were generated using biotinylated or non-biotinylated forward primer and non-biotinylated reverse primers with Platinum Taq PCRx DNA Polymerase (Invitrogen) and amplification conditions: initial denaturation at 95°C for 2 min., followed by 35 cycles of denaturation at 95°C for 30 sec., annealing at 60°C for 30 sec. and extension at 68°C for 1 min., and final extension at 68°C for 6 min. The mouse *Nags* (m*Nags*) promoter regions A and B (Figure 1) were amplified with primer pair mNAGS-Prom Region A, from +97 to −259, relative to the translation initiation codon and with mNAGS-Prom Region B, from −302 to −776, respectively (Table S3). A region of m*Nags*, that is not highly conserved in mammals, −1056 to −1320, was amplified using primer pair mNAGS-Prom-NC to serve as a negative control for the promoter regulatory region. The enhancer region of mNAGS, spanning from −2834 to −3167, was amplified using forward primer pair mNAGS-enh. The negative control for the enhancer region, a non-conserved region located close to enhancer, was the amplification product of primer pair mNAGS-Enh-NC spanning −5569 to −5997 upstream of m*Nags*. Additional negative controls, non-biotinylated probes, were generated using each primer pair.

#### Preparation of nuclear extracts

Nuclear extract was isolated from donated adult mouse livers of C57BL/6 mice using Nuclear Extraction Kit (Origene) according to manufacturer's instructions. The protein concentration of the nuclear extract was determined using bovine serum albumin as the protein standard with Bradford Assay dye concentrate reagents (Bio-Rad). On average, 10 mg of nuclear protein was obtained from mouse liver.

#### Binding Protocol and Western Blot

For the avidin-agarose protein-DNA pull-down assay [52], 1 mg of nuclear extract in PBS buffer containing inhibitors (PBSI; 1× PBS with 0.5 mM PMSF, 25 mM β-glycerophosphate, mM NaF), 15 ug of DNA probe, and avidin-agarose beads (Sigma) were combined and incubated for 16 hrs on a rotating shaker at 4°. The probe and bead concentrations were in excess to ensure complete pull-down of DNA–protein complexes. Following incubation, the supernatant was reserved while the beads were washed 3 times with cold PBSI and then resuspended and boiled in Laemmli protein denaturing buffer (Bio-Rad) with 0.2 M DTT. The supernatant was also combined with denaturing buffer with DTT and boiled; all samples were loaded onto 10% SDS–polyacrylamide gel. The proteins were separated by electrophoresis, transferred to a nitrocellulose membrane, and then identified by immunoblotting using primary antibodies at 1∶2000 dilution of antibody to Sp1 (Santa Cruz Biotech; Millipore), 1∶1000 dilution of CREB-1α/β (Santa Cruz Biotech), and 1∶3000 dilution of C/EBPα/β (Santa Cruz Biotech) for the promoter region and 1∶500 dilution of HNF-1α/ß (Santa Cruz Biotech), 1∶1000 dilution of NF-Yα (Santa Cruz Biotech) and 1∶2000 dilution of SMAD2/3 (Santa Cruz Biotech) for the −3 kb conserved region. The membrane was than incubated with 1∶20,000 dilution of donkey anti-rabbit secondary antibody conjugated to horseradish peroxidase (Pierce) and bands were visualized using SuperSignal West Pico Kit (Pierce) according to manufacturer's instructions.

### Chromatin Immunoprecipitation

#### Tissue preparation and DNA immunoprecipitation

Donated livers from adult C57BL/6 mice were minced and chromatin was precipitated using SimpleChIP Enzyme Chromatin Kit (Origene) with the variation for whole tissue. Briefly, fresh tissue was minced and washed with PBS including Protease Inhibitor Complete tablets (Roche). Proteins and DNA were cross-linked with 1.5% formaldehyde, and tissue was disaggregated with dounce homogenizer. Chromatin was sheared to an approximate size of 100–1000 bp by micrococcal nuclease digestion followed by sonication. Immunoprecipitation was conducted using antibodies to transcription factors Sp1 (Millipore), CREB (Santa Cruz Biotech), C/EBP (Santa Cruz Biotech), HNF-1 (Santa Cruz Biotech), NF-Y (Santa Cruz Biotech), SMAD2 (Santa Cruz Biotech) and AP-2 (Santa Cruz Biotech) and control antibodies to histone H3 and non-specific rabbit IgG (Cell Signaling Technologies). Chromatin was eluted from protein G agarose beads, cross-linking was reversed, and DNA was purified according to manufacturer's instructions.

#### Real-time PCR quantification

ChIP enriched DNA samples included 2% input control and dilutions for a standard curve, positive control immunoprecipitate from anti-histone H3 antibody sample, negative control immunoprecipitation from anti-rabbit IgG antibody, no antibody control, water control, and test antibodies. Enriched DNA was subject to quantitative real-time PCR using iTaq SYBR Green Supermix with ROX (Bio-Rad) and gene specific primers (Table S4) including negative locus primers to Chemokine ligand 2 (MIP-2) on a 7900HT Fast Real-Time PCR System (Applied Biosystems). Amplification conditions included initial denaturation at 95°C for 2 min., followed by 50 cycles of denaturation at 95°C for 30 sec., annealing at 60°C for 30 sec. and extension at 72°C for 30 sec., with dissociation steps of 95° for 15 sec. followed by 50° for 15 sec. and finally 95° for 15 sec. Samples were amplified and analyzed using 7900HT Sequence Detection System Software (Applied Biosystems). Values were expressed as mean ± SEM and analyzed using Student's *t*-test.

## Results

### Selected regions of non-coding DNA upstream of NAGS are highly conserved

15 kilobase of genomic DNA sequence 5′ of the translational start site of *NAGS* and sequence of the first intron from human, chimpanzee, dog, horse, cow, mouse and rat were aligned and compared using pair-wise BLAST. Comparisons showed three highly conserved regions upstream of human *NAGS* at −57 to −284, −498 to −576, and −2978 to −3344 relative to the start ATG, and no significant conservation within the intron or between −5 and −15 kb upstream (Figure 1). The region within −1 kb of the translational start site was designated as the putative promoter while the region 3 kb upstream was designated a putative regulatory element. Figure 1 also shows an alignment of mammalian *NAGS* genes using phastCons (green) and phyloP (blue), which identified three non-coding regions of conservation located 3 kb upstream, immediately upstream, and within the first intron of *NAGS*, respectively (Figure 1). The phastCons, phyloP and our analyses of conservation within the NAGS gene differed due to different algorithms that were used to identify regions of conservation [39], [53], [54].

To validate our strategy for identification of conserved regions, the same analyses were conducted for *CPS1*, a gene in which a proximal promoter and an enhancer element located 6.3 kb upstream of rat *Cps1*, have been characterized [55], [56], [57]. 15 kb of *CPS1* genomic DNA sequence 5′ of the translational start site was collected from human, chimpanzee, dog, mouse and rat and compared using pair-wise BLAST. Five regions of high conservation were identified including the previously reported proximal promoter located immediately upstream of the translation initiation codon and the enhancer at −7392 to −7966 relative to ATG of the human *CPS1* gene (Figure S1). In addition, three previously unknown regions, termed A, B and C, were also identified at −5, −10.5 and −12 kb relative to *CPS1* translation initiation codon (Figure S1). PhastCons and phyloP alignment of mammalian genomic DNA identified the same 5 conserved regions (Figure S1).

### Highly conserved, non-coding regions of NAGS function as promoter and enhancer elements for gene transcription

**Figure 2:**
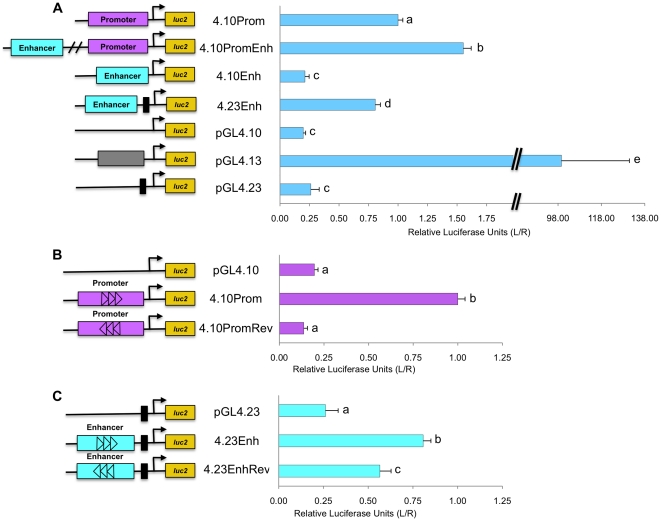
Highly conserved regulatory regions, upstream of the *NAGS* gene, function as promoter and enhancer elements. In liver derived cells the *NAGS* promoter (4.10Prom), promoter+enhancer (4.10PromEnh), enhancer with TATA promoter (4.23Enh), and positive control promoter vector (pGL4.13) significantly simulate transcription while the enhancer (4.10Enh), basic vector (pGL4.10) does not stimulate transcription above baseline (A). Reverse insertion of the promoter (4.10PromRev) did not stimulate transcription compared to 4.10Prom and pGL4.10 vector (B), but reverse enhancer (4.23EnhRev) significantly stimulated transcription compared to 4.23Enh and pGL4.23 vector (C). Calculated results are an average of three independent experiments that were each carried out in triplicate, normalized to *Rluc* expression, and expressed relative to the promoter for each experiment with error reported as ±SEM. Lowercase letters indicate statistically significant differences.

Reporter assays were used to examine the functionality of each of the following: wild type *NAGS* promoter (4.10Prom), control reversed promoter (4.10PromRev), enhancer alone (4.10Enh), promoter and enhancer (4.10PromEnh), and enhancer in both orientations with the heterologous TATA-box promoter (4.23Enh and 4.23EnhRev) by measuring the expression of a luciferase reporter gene in cultured HepG2 cells (Figure 2A). Vectors pGL4.13, pGL4.23, and pGL4.10 containing firefly luciferase *luc2*, with an SV40 promoter, a minimal TATA-promoter, or without a promoter respectively, were used as positive, baseline reference, and negative assay controls. Vector pGL4.74, containing *Renilla* luciferase *hRluc*, was co-transfected with each plasmid to control for transfection efficiency.

The human *NAGS* promoter alone (plasmid 4.10Prom), stimulated transcription of the luciferase gene while the upstream regulatory region (plasmid 4.10Enh) alone, did not (Figure 2A). When the *NAGS* promoter and upstream regulatory region were both present (4.10PromEnh plasmid), transcription increased by 50% compared to the promoter alone confirming that the upstream conserved region can function as an enhancer of transcription. When the *NAGS* enhancer was paired with a heterologous promoter containing a TATA-box, in the 4.23Enh construct, the transcription of luciferase about three times higher compared to construct with minimal TATA-box. The backbone vector 4.10 did not stimulate expression of the luciferase gene. As expected, positive control vector 4.13, containing a strong promoter, activated transcription in this cell culture system (Figure 2A). The promoter in the reverse orientation (4.10PromRev) did not activate luciferase expression indicating that the *NAGS* promoter acts in a direction dependent manner (Figure 2B). The ability of the *NAGS* enhancer (4.23EnhRev) to stimulate transcription with the heterologous promoter was orientation independent (Figure 2C). Similar results were obtained for reporter assays using mouse promoter and enhancer (Figure S2).

### Transcription of NAGS initiates at multiple sites

**Figure 3:**
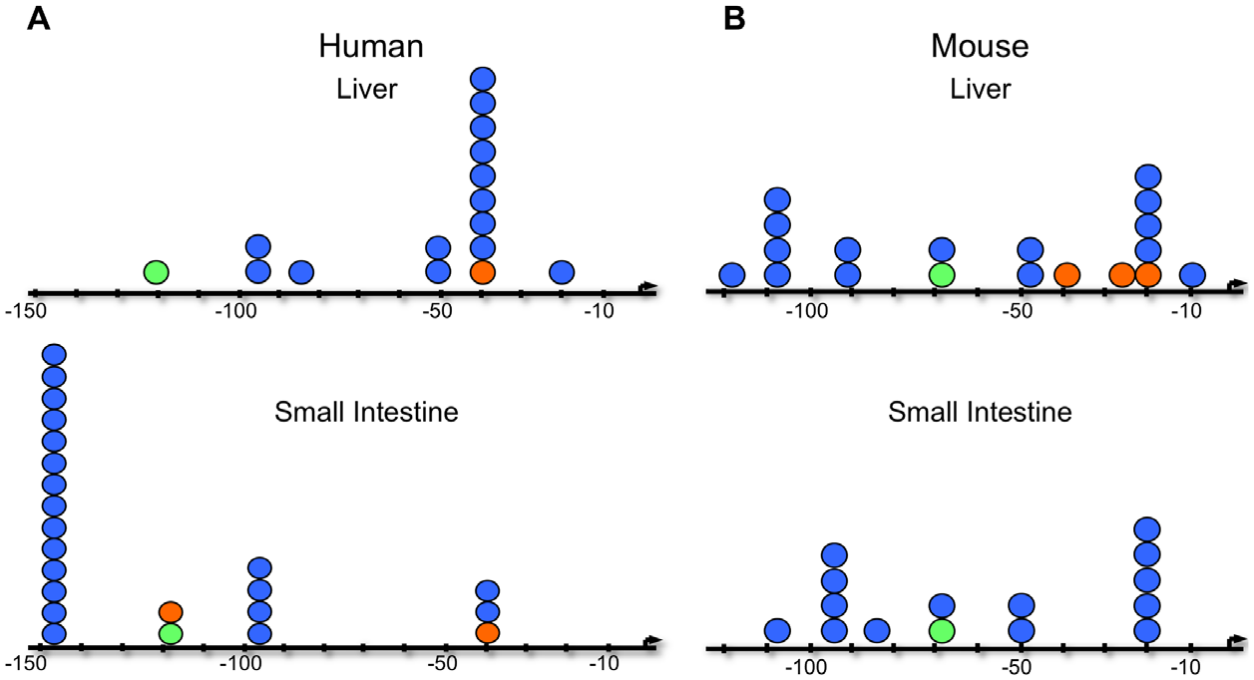
Transcription start sites (TSS) are species and tissue specific. TSS identified in the promoter of *NAGS* by 5′RACE analysis (blue circles), the Database of Transcriptional Start Sites (DBTSS) (green circles) and 5′ termination sites of Expressed Sequence Tags (ESTs) from Genbank (orange circles) were aligned on the DNA sequence 5′ of the human (A) and mouse (B) *NAGS* coding sequence. The arrow indicates the translation start site.

Following discovery of the *NAGS* promoter, the transcriptional start sites (TSS) in human and mouse liver and small intestine were identified using 5′ RACE (Figure 3A and B). Cloned and sequenced amplification products from 5′RACE were aligned along the 5′ non-coding region of *NAGS* along with TSS identified in the Database of Transcriptional Start Sites (DBTSS) and expressed sequence tags (ESTs) from Genbank. Results suggest that *NAGS* has multiple TSS and that some may be species and tissue-specific. Combined 5′RACE, DBTSS, and Genbank results indicate that within human liver, the most frequently occurring TSS was at −42 bp upstream of the ATG codon, while in human small intestine it was at −146 bp (Figure 3A). Within mouse tissues, no dominant TSS was evident, but transcription of the *NAGS* gene initiated most often from −20 bp and −108 bp in liver and −20 bp and −95 bp in small intestine (Figure 3B). Figure 3 also shows several other rare TSS that were identified.

### Transcription factors bind highly conserved motifs within the promoter and enhancer of NAGS

**Figure 4:**
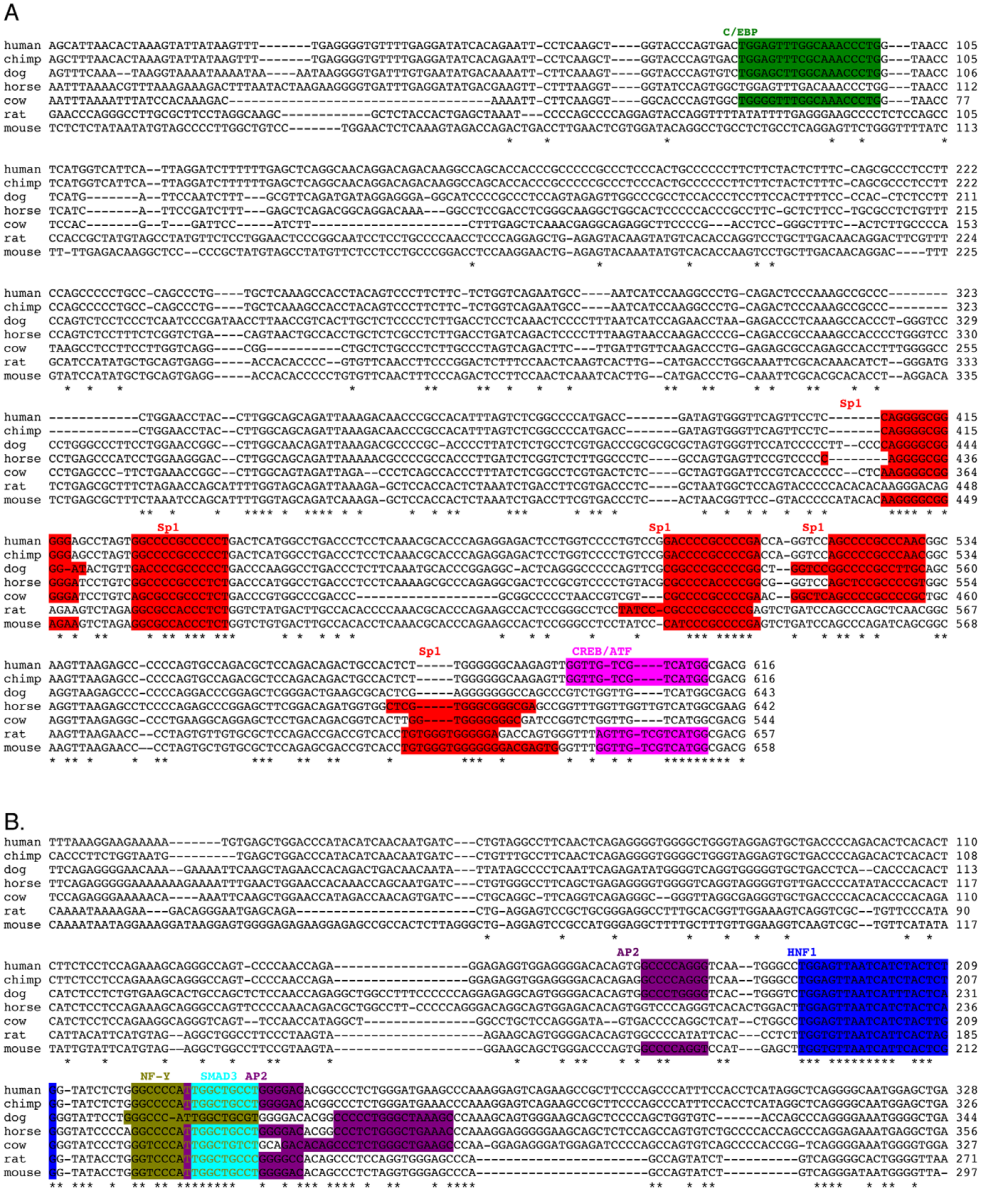
Sequence alignment of *NAGS* promoters and enhancers from seven mammalian species indicate conserved motifs. DNA sequence of the promoter (A) and enhancer (B) regions were aligned using CLUSTALW alignment software. CLOVER analysis was used to identify transcription factor binding motifs. Binding sites for C/EBP (green), Sp1 (red), CREB/ATF (pink), AP-2 (purple), HNF-1 (blue), NF-Y (olive), and SMAD 3 (cyan) were highly conserved.

When promoters and enhancers from six mammalian NAGS genes were aligned, there were multiple regions of base pair conservation (Figure 4). Cis-eLement OVER-representation (CLOVER) software analysis was employed to identify transcription factor binding motifs in regulatory regions of human, chimpanzee, horse, cow, dog, mouse, and rat *NAGS*. Analyses of the region +9 to −996 bp (relative to the translational start codon, promoter, Table S6) and −2866 to −3620 bp (enhancer, Table S7) predicted several transcription factor binding motifs that are expressed in the liver, but no TATA-box for transcription initiation. Sp1 binding sites, within the promoter, and the HNF-1 binding motif, within the enhancer, received the highest over-representation scores, but additional motifs with lower scores were also over-represented.

**Figure 5:**
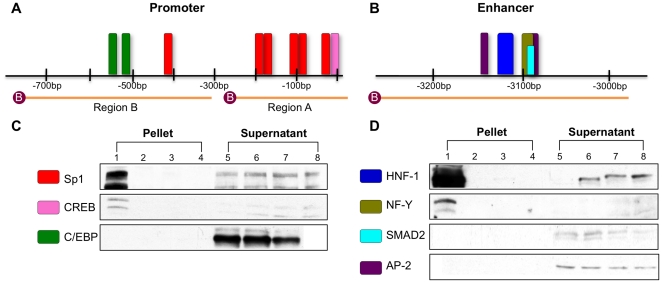
DNA-protein avidin-agarose pull-down assay results confirm transcription factor binding. Two probes for the promoter (A) and one probe for the enhancer (B) encompass the highly conserved transcription factor binding motifs of *NAGS*. The motif colors reflect the colors used in figures 4A and B. Assays followed by immunoblot confirmed binding of Sp1 and CREB, but not C/EBP within the promoter (C) and HNF-1 and NF-Y, but not SMAD3 or AP-2 within the enhancer regions (D). Lanes 1–4 represent precipitated proteins from mouse liver nuclear extract bound to biotinylated probes of the regions of interest (Lane 1), non-biotinylated probes of the regions of interest (Lane 2), biotinylated probes of non-specific regions (Lane 3), and no probe (Lane 4). Lanes 5–8 represent supernatant fluid from overnight incubation of biotinylated probes of the region of interest (Lane 5), non-biotinylated probes of the region of interest (Lane 6), biotinylated probes of the non-specific regions (Lane 7), or no probe (Lane 8). Immunoblots are representative of at least three replicate experiments.

Next, over-represented motifs were mapped on the CLUSTALW alignments (Figure 4A and 4B) and motifs with high conservation, having been identified in at least four out of the seven mammalian species, were examined further. Throughout the promoter, five binding sites for Sp1 were highly conserved, two of which were conserved in all examined species. A binding site recognized by CREB and Activating Transcription Factor-1 (ATF-1) was conserved in four species and overlapped with the translation start codon; a C/EBP binding site was identified farther upstream in region B of the promoter (Figures 4A & 5A). Within the enhancer, a binding site for HNF-1 was conserved in all species. Overlapping binding sites for NF-Y, AP-2 and Mothers Against Decapentaplegic Homolog 3 (SMAD3) were also conserved in all species, while an additional AP-2 binding site, located 5′ of the HNF-1 site, was conserved in four out of seven species (Figure 4B & 5B).

To validate computational strategy for identification of transcription factor binding sites, the enhancers of human, chimpanzee, dog, mouse, and rat *Cps1* were analyzed using CLOVER, and the experimentally identified binding motifs for C/EBP, CREB, GR, AP-1 and HNF-3 [55], [56], [57] were detected along with additional unreported motifs for HNF-4, AR, C/EBP and HNF-3 (Figure S3, Table S5). The detection of experimentally confirmed binding motifs in *CPS1* has made the use of CLOVER for bioinformatic analysis of *NAGS* credible.

A DNA-protein pull-down assay was devised to test the bioinformatic prediction of specific binding sites. Two biotin-labeled DNA probes for the promoter (Figure 5A) encompassed regions A and B (Lane 1 in Figure 5C) and one probe (Figure 5B) encompassed the enhancer (Lane 1 in Figure 5D). A biotinylated probe to a region upstream of the *NAGS* gene, lacking any highly conserved motifs (Lane 3 in Figures 5C and 5D), and non-biotinylated probes to region A or B (Lane 2 in Figures 5C and 5D) were used as negative controls. The supernatant fluid from each pull-down was included as a positive control for the presence of the transcription factor (Lanes 5–8). Intensities of bands corresponding to each transcription factor in supernatant fluids were also used as indicators of pull-down efficiency.

Factors Sp1 and CREB bound to the probe of promoter region A (Lane 1 in Figure 5C). Sp1 also bound to the probe of promoter region B (data not shown) while C/EBP did not bind to this probe (Lane 1 in Figure 5C). Within the enhancer region, transcription factors HNF-1 and NF-Y bound to the probe, however SMAD2/3 and AP2 did not (Lane 1 in Figure 5D). Binding of Sp1, CREB, C/EBP, HNF-1, NF-Y, SMAD2/3, and AP-2 was not detected in the negative controls (Lanes 2–4 in Figures 5C and 5D) while each transcription factor was detected in the positive controls of liver nuclear extract supernatants (Lanes 5–8 in Figures 5C and 5D). Each immunoblot result is representative of at least three replicate experiments.

**Figure 6:**
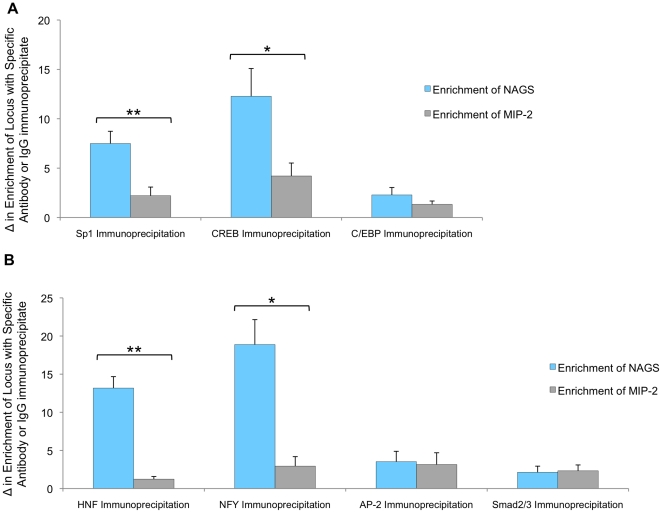
Chromatin Immunoprecipitation (ChIP) results confirm transcription factor binding. ChIP with transcription factor antibodies was compared to negative control IgG antibody. Real-Time PCR using promoter or enhancer specific primers was compared to primers for the negative locus MIP-2. The results confirmed that Sp1 and CREB but not C/EBP bind within the promoter (A) and HNF-1 and NF-Y but not AP-2 or SMAD2/3 bind within the enhancer region (B) of *NAGS*. Calculated error was from three replicate experiments and reported as ± SEM. One asterisk (*) indicates p<0.05 and two asterisks (**) indicate p<0.005.

Binding of transcription factors to the predicted motifs was also confirmed using chromatin immunoprecipitation (ChIP) followed by Real-Time PCR. Measurement compared the enrichment of target DNA regions to the negative control locus MIP-2. ChIP with Sp1 and CREB antibodies significantly enriched the *NAGS* promoter DNA compared to MIP-2 (p<0.005 and p<0.05, respectively; Figure 6A). ChIP with C/EBP antibody did not enrich the *NAGS* promoter DNA compared to the negative locus (p>0.05; Figure 6A). The *NAGS* enhancer was enriched in chromatin immunoprecipitated with antibodies against HNF-1 and NF-Y (p<0.005 and p<0.05, respectively; Figure 6B), but not with antibodies against AP-2 and SMAD2/3 (p>0.05; Figure 6B). Thus, Pull-down and ChIP assays confirmed that Sp1 and CREB bind along the *NAGS* promoter and HNF-1 and NF-Y bind along the enhancer.

### Transcription factors and binding motifs are functionally important for transcription

**Figure 7:**
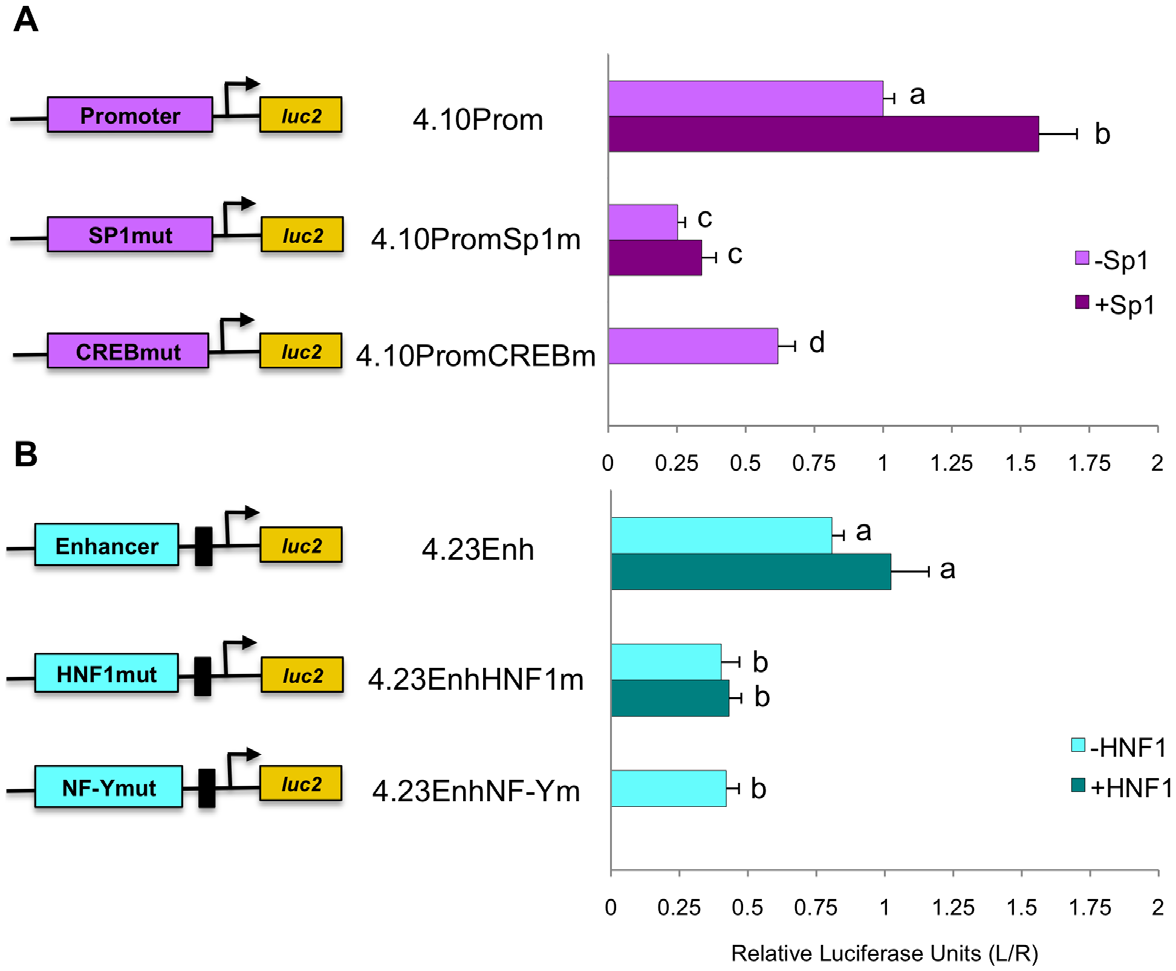
Transcription factors Sp1, CREB, HNF-1, and NF-Y are functionally important for stimulating expression of reporter gene transcription. Mutagenesis of the putative transcription factor binding sites significantly decreases transcription by the promoter (A) and the enhancer with TATA promoter (B) in liver derived cells when compared to non-mutated sites. Addition of Sp1 with the promoter (A) and HNF-1 with the enhancer (B) increases transcription driven by non-mutated constructs. Calculated results are an average of three independent experiments that were each carried out in triplicate, normalized to *Rluc* expression, and expressed relative to the promoter for each experiment with error reported as ±SEM. Lowercase letters indicate statistically significant differences.

Reporter assays in liver hepatoma cells with mutated transcription factor binding motifs demonstrate the functional importance of each site. Following these sequence substitutions, transcription factor binding motifs were no longer detected by CLOVER (Table 2). Within the promoter, point mutations in the Sp1 binding sites decreased the expression of reporter gene by 75% (p<0.005) and point mutations in the CREB binding site resulted in a 40% decrease (p<0.005; Figure 7A). Point mutations in the HNF-1 or NF-Y binding sites, in the enhancer, decreased expression of luciferase reporter by 50% (p<0.005 for both; Figure 7B).

While these results confirm that each motif is important for transcription, the functional importance of Sp1 and HNF-1 proteins is demonstrated by co-expression of the proteins with reporter assay constructs. Co-transfection of Sp1 expression plasmid with the *NAGS* promoter (4.10Prom) increases expression of luciferase more than 50% (P<0.005; Figure 7A) while co-transfection of HNF-1 expression construct with the enhancer and minimal TATA promoter (4.23Enh), increases expression of the reporter gene by 25% (p>0.05; Figure 7B) suggesting that endogenous Sp1 and, less so, HNF-1 do not saturate their binding motifs on the transfected reporter plasmids.

**Figure 8:**
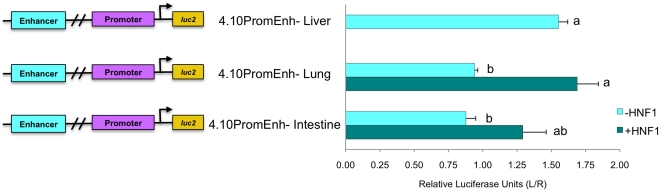
The *NAGS* enhancer shows tissue specificity. The enhancer with *NAGS* promoter (4.10PromEnh) increases transcription relative to the promoter in liver derived cells but not in intestine or lung derived cells (cyan bars) without the addition of HNF-1 protein (teal bars). Calculated results are an average of three independent experiments that were carried out in triplicate, normalized to *Rluc* expression, and expressed relative to the promoter for each experiment with error reported as ±SEM. Lowercase letters indicate statistically significant differences.

Reporter assays to compare the effect of the enhancer in liver, intestine and lung cells, included data that were normalized to the reporter expression driven by the NAGS promoter. While the *NAGS* enhancer (4.10PromEnh) increased expression of the reporter gene by 50% in liver derived cells (Figure 2A), expression of the luciferase gene did not increase in the intestine or lung derived cells (Figure 8) suggesting that the enhancer may determine tissue specificity of NAGS expression. When HNF-1 expression plasmid and 4.10PromEnh were co-transfected into intestine and lung derived cells, transcription was stimulated to levels that were not significantly different from 4.10PromEnh in liver cells (p>0.05) (Figure 8). Because intestine and lung derived cells lack HNF-1 (data not shown), this demonstrated the importance of HNF-1 and NAGS enhancer for the tissue specificity of NAGS expression.

## Discussion

In this study we used bioinformatic analyses to predict regulatory regions based on the hypothesis that non-coding DNA sequences that are highly conserved between species are important for gene regulation. Multiple pair-wise BLAST alignments and sequence alignment from the UCSC genome browser were used to identify two conserved regions within *NAGS*, which were determined to be a promoter and an enhancer. The efficacy of this method was confirmed by successful identification of the experimentally identified promoter and −6.3 kb enhancer [25], [31], along with three additional highly conserved regions, in the non-coding region upstream of *CPS1*. It should be noted that the high stringency of our BLAST analysis (80% identity and at least 100 bp of aligned sequence in four or more species) was selected to identify conserved regions that could support multiple binding sites where complexes of transcription factors may form [25], [58]. This may have caused us to overlook species specific or isolated binding motifs, such as the recently identified FXR binding site [59].

The reporter assay results confirm that the two highly conserved regions within 1 kb and 3 kb upstream of the translational start site function as promoter and enhancer, respectively. The promoter activates expression of the luciferase reporter gene and we therefore infer that it will activate transcription of *NAGS in vivo*. Similarly, the enhancer in either orientation increases expression of luciferase by approximately 50% relative to the promoter alone, suggesting that it stimulates *NAGS* transcription as well. The relatively small but significant effect of the enhancer could be due to spacing differences between the genomic *NAGS* promoter and enhancer and their spacing in the reporter constructs. Alternatively, while HepG2 cells express transcription factors that we identified using bioinformatic tools, the *NAGS* enhancer may bind additional factors, absent in HepG2 cells, and have larger effect *in vivo* than in cultured cells. Another explanation for the relatively small effect of the *NAGS* enhancer is the possible presence of a proximal enhancer within the region we termed the promoter. Additional experiments are necessary to distinguish between these two possibilities.

Our analysis of the *NAGS* transcriptional start sites identified multiple TSS that may be species and tissue specific. While the function of each TSS is unknown, these results are consistent with transcription initiation by Sp1 [16], [60], [61], and future experiments may find that they are involved in transcriptional control for tissue specific expression, developmental-stage specific expression, quantitatively different levels of mRNA expression, or may even determine the transcript stability [62].

After we confirmed that the promoter and enhancer initiate and increase transcription, we looked for transcription factors that bind and regulate *NAGS* in these regions. By filtering for the highly over-represented and spatially conserved binding sites, relative to the translational start codon, we identified Sp1, CREB, and C/EBP in the promoter and HNF-1 AP-2, NF-Y, and SMAD-3 in the enhancer as transcription factors that could bind to the *NAGS* upstream region. This filtering method was confirmed by analysis of the −6.3 kb enhancer of *CPS1* in which binding sites for the previously published C/EBP, CREB, GR, and HNF-3 were identified.

The protein-DNA pull down assays, designed to test which transcription factors among a pool of nuclear proteins bind to amplified sequence of conserved upstream DNA, confirmed that Sp1, CREB, HNF-1 and NF-Y bind to *NAGS* promoter and enhancer, while we could not detect binding of C/EBP, AP-2 and SMAD3 (Figure 5). We initially used 60 bp probes encompassing a specific binding motif for the protein–DNA pull down assays. However, probes encompassing the entire region were better able to bind transcription factors (data not shown), suggesting that binding is facilitated by interactions with DNA sequences outside predicted binding sites and possibly other transcription factors and co-activators. ChIP analysis was used to confirm binding of the predicted transcription factors to the DNA regions of interest under physiological conditions. ChIP and DNA-pull down assays confirmed that Sp1 and CREB bind to the promoter and HNF-1 and NF-Y bind to the enhancer of *NAGS* (Figures 5 and 6), while reporter assays demonstrated the functional importance of each binding motif by a decrease in transcription following mutagenesis of the binding sites (Figure 7).

Furthermore, we have demonstrated that Sp1 and HNF-1 are important for stimulation of transcription of *NAGS* and that HNF-1 determines tissue specificity of *NAGS* expression. In the liver derived cell line, co-transfection of either Sp1 or HNF-1 expression plasmids with reporter constructs containing the *NAGS* promoter and enhancer led to increased expression of the reporter gene (Figure 7) suggesting that these two transcription factors regulate expression of *NAGS* in the liver. In the lung and intestine derived cell lines, expression of HNF-1 was sufficient to activate expression of reporter gene in constructs containing *NAGS* enhancer and promoter (Figure 8). This suggests that HNF-1 binding to the *NAGS* enhancer determines tissue specificity of NAGS expression. Testing the effect of over-expression of CREB protein was hindered by its capacity to homo- and heterodimerize with multiple partners [63], [64]. The effect of NF-Y was not tested because this transcription factor is a heterotrimer [65] and its co-expression with reporter plasmids would require stable expression of NF-Y subunit proteins by *in vitro* cell culture before reporter plasmids can be transfected and assayed for NF-Y effect on transcription.

From the data provided herein, we can speculate on the potential role these factors play in regulating *NAGS* transcription. First, in the absence of a canonical TATA-box, transcription initiated by Sp1 often results in multiple transcriptional start sites [66], [67]. Sp1 is a strong activator of transcription [16], [68], [69], [70], [71] and when multiple Sp1 sites are present, as in *NAGS*, multiple Sp1 proteins can form complexes with each other and synergistically activate transcription [16], [69]. Because transcription is significantly increased by co-expression with Sp1 protein and decreased following mutation of the Sp1 binding sites, Sp1 may prove to be the activator of *NAGS* transcription, similar to its role for *ASS*, *ASL* and *ARG1* [15], [25].

Second, studies have shown that glucagon and second messenger cAMP trigger a cascade that phosphorylates CREB and allows for DNA binding and activation of transcription [72], [73]. In *CPS1* and *ASS*, CREB stimulates transcription upon glucagon signaling [15], [31]. Decrease in transcription following CREB mutation and the close proximity of Sp1 and CREB binding sites among the TSS suggests that the transcription initiation machinery may be recruited by these factors, and future research should examine this postulate.

Our experiments and other studies [74] confirm the role of HNF-1 in *NAGS* expression. HNF-1 is essential for stimulation of NAGS expression by its enhancer. This factor is in part regulated by HNF-3, HNF-4, and C/EBP, each of which are known to regulate other urea cycle genes [75], [76], [77]. Future research will focus on the mechanism of control between these factors, HNF-1, and *NAGS*. Our study has also shown that NF-Y is an activator of *NAGS* expression, and future studies will focus on the exact mechanism of its function in this context.

The human NAGS gene on the forward strand of chromosome 17 partially overlaps with the peptide YY (*PYY*) gene, which is on the reverse strand. This overlap was identified with a *PYY* cDNA isolated from a brain astrocytoma cDNA library that has an 80 nucleotide long exon located between regions A and B of the *NAGS* promoter [78], [79] (Figure 1). Other full-length *PYY* transcripts initiate about 500 bp upstream of the *PYY* coding region, which is located 51 kb upstream of the *NAGS* translation initiation codon. Recent analysis of human transcripts revealed that many protein coding loci are associated with at least one transcript that initiates from a distal site [80], but the significance or function of these transcripts remains to be elucidated. Partial overlap between human *NAGS* and *PYY* genes raises the interesting possibility that these two genes share *cis*-acting regulatory elements and might be co-regulated [79], [81]. The mechanism of co-regulation of human *NAGS* and *PYY* is likely to be complex because of their differing tissue expression patterns [1], [82], [83], [84] including different cell types within the intestine. *PYY* is expressed in the intestinal neuroendocrine cells [85], [86] while epithelial cells in the small intestine express *NAGS* [87], [88], together with *OTC* and *CPS1* [13], [89]. Inspection of the transcription factor binding track of the UCSC genome browser revealed two binding sites for the CTCF transcription repressor between *NAGS* and *PYY* genes; they are located approximately 9.5 and 21 kb upstream of the *NAGS* coding region. The CTCF binding sites could act as chromatin insulators [90], [91], [92] and either block regulation of *PYY* by the *NAGS* enhancer or enable cell type specific regulation of each gene by the *NAGS* enhancer and promoter. Our results show that the *NAGS* promoter in the reverse orientation does not activate transcription of the reporter gene in liver derived cells (Figure 2), but this does not preclude transcription activation in other cell types, not tested in this study. It is possible that the *NAGS* promoter, enhancer, or other *NAGS* regions, regulates expression of *PYY* [84], and reporter assays in tissues and cultured cells which express *PYY* would test this hypothesis.

While regulation of *NAGS* by Sp1, CREB, HNF-1, NF-Y, and factors that regulate them, requires additional study, identification of regions that regulate human *NAGS* and *OTC* have enabled diagnosis of patients with clinical symptoms of urea cycle disorders, but lacking disease causing mutations in the coding regions of the genes [93], [94]. Recently, we identified a patient with a mutation in the enhancer of *NAGS* and confirmed the diagnosis of NAGS deficiency by showing that the mutation significantly decreases transcription of *NAGS* [93]. This example suggests that identification of regulatory regions within genes will lead to more and better diagnoses of urea cycle disorders and other genetic diseases and to accurate genetic counseling.

In conclusion, this study identified a promoter and a tissue specific enhancer of *NAGS* and functionally relevant transcription factor binding motifs within these regions. The results show that Sp1 and CREB bind to the *NAGS* promoter, suggesting that glucagon and cAMP signaling may regulate the expression of *NAGS*. Within the enhancer, HNF-1 may be an important factor in the coordinated regulation of this urea cycle gene transcription through its interaction with HNF-3, HNF-4 and C/EBP while the role of NF-Y is less clear considering that NF-Y may function as an activator or repressor. While additional studies will be needed to further define the roles of these factors, these results contain the first thorough analysis of NAGS and suggest networks of control between signaling cascades, *NAGS* and the coordinated regulation of the other urea cycle genes.

## Supporting Information

Figure S1 **Regions Upstream of mammalian** ***CPS1*** **genes are highly conserved.** Three new highly conserved regions were identified within 15 kb 5′ of the *CPS1* translational start site. Conservation algorithms phastCons (green) and phyloP (blue) from the UCSC genome browser indicate regions that are highly conserved across all mammals (A). Pair-wise blast analysis of human, chimpanzee, dog, mouse, and rat 5′ non-coding region of *CPS1* were used to identify two known and three previously unknown regions of high conservation, referred to enhancer/repressor regions A, B, and C. Highly conserved regions within the *CPS1* 5′ non-coding sequence include the proximal promoter, region A, the -enhancer, region B, and region C.
(TIF)

Figure S2 **Highly conserved regulatory regions, upstream of the mouse** ***Nags*** **gene, function as promoter and enhancer elements.** Mouse promoter (m4.10Prom), promoter and enhancer (m4.10PromEnh), and enhancer with TATA promoter (m4.23Enh) stimulated transcription while enhancer lacking a promoter (m4.10Enh) did not in liver cells. Calculated results are an average of three independent experiments that were carried out in triplicate, normalized to *Rluc* expression, and expressed relative to the promoter for each experiment with error reported as ±SEM.
(TIF)

Figure S3 **Novel transcription factor binding motifs, in the enhancer region of** ***CPS1*** **, were identified using CLOVER.** Several highly conserved transcription factor binding sites were present in the enhancer region. An asterisk denotes an experimentally verified transcription factor binding site. All motifs were spatially conserved between mammalian species.
(TIF)

Table S1 **Sequences of primers that were used to amplify human or mouse DNA by PCR for insertion of the promoter and enhancer regions into sequencing and reporter assay vectors.**
(DOCX)

Table S2 **Primer sequences used to determine transcription start sites of** ***NAGS*** **with 5′ RACE.** Primers were designed according to manufacturer's instructions and used to determine transcription start sites of human and mouse *NAGS* in liver and small intestine RNA using 5′ RACE.
(DOCX)

Table S3 **Primer sequences used to generate DNA probes of the specified regions of m** ***Nags*** **.** Primers were used to generate DNA probes, by PCR, of the promoter, enhancer, or non-specific specified regions of m*Nags*.
(DOCX)

Table S4 **Primer sequences used for quantitative real-time PCR analysis of chromatin immunoprecipitation samples.**
(DOCX)

Table S5 **Results of CLOVER analysis of the enhancer region with sequence information for human and mouse** ***CPS1*** **.** Results were filtered to exclude motifs for transcription factors that are not expressed in the liver.
(DOCX)

Table S6 **Results of CLOVER analysis of the promoter region with sequence information for human and mouse** ***NAGS*** **.** Results were filtered to exclude motifs for transcription factors that are not expressed in liver.
(DOCX)

Table S7 **Results of CLOVER analysis of the enhancer region with sequence information for human and mouse** ***NAGS*.** Results were filtered to exclude motifs for transcription factors that are not expressed in the liver.
(DOCX)
