# Supplementary figures and images for: Transcriptional Regulation of N-Acetylglutamate Synthase
Source: PLoS One. 2012 Feb 27;7(2):e29527. doi: 10.1371/journal.pone.0029527 (PMC3287996; doi:10.1371/journal.pone.0029527)

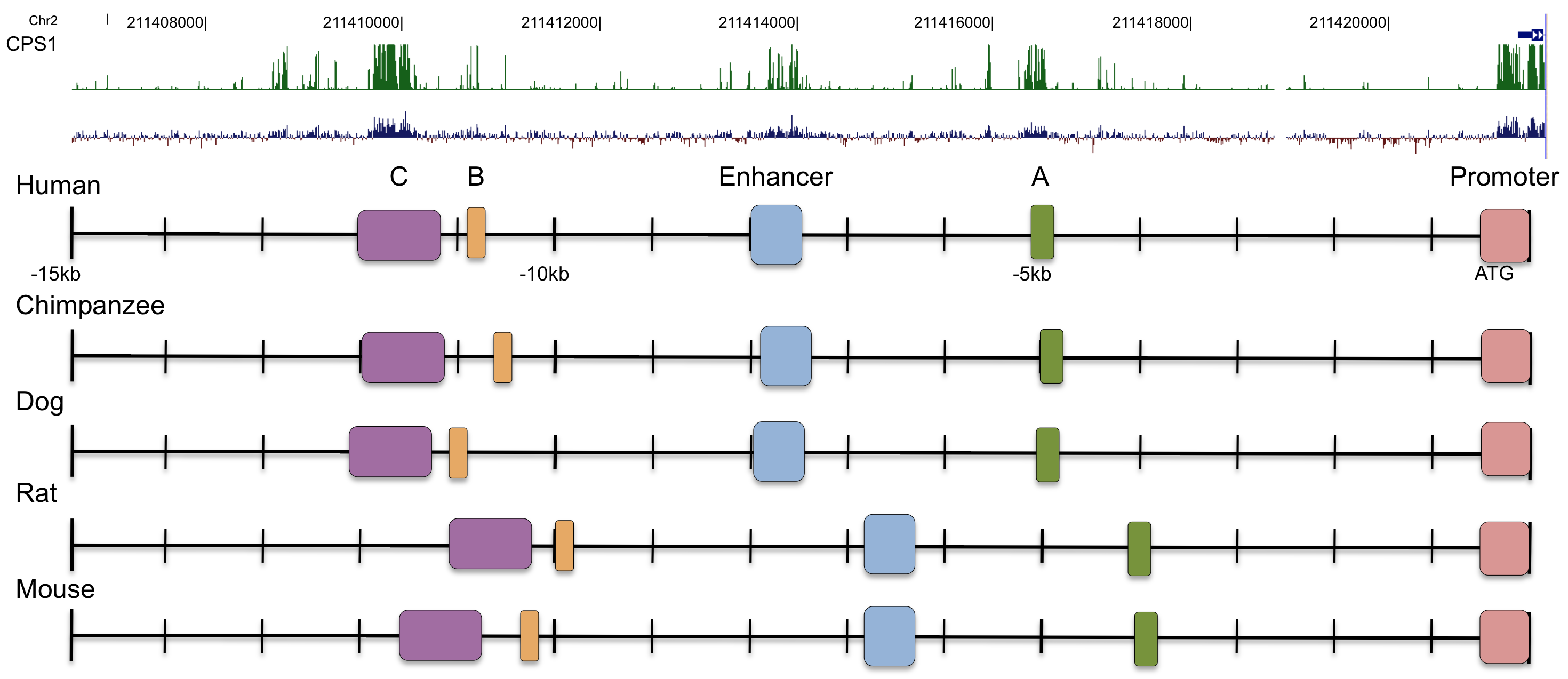

Supplement: Figure S1 — Regions Upstream of mammalian CPS1 genes are highly conserved. Three new highly conserved regions were identified within 15 kb 5′ of the CPS1 translational start site. Conservation algorithms phastCons (green) and phyloP (blue) from the UCSC genome browser indicate regions that are highly conserved across all mammals (A). Pair-wise blast analysis of human, chimpanzee, dog, mouse, and rat 5′ non-coding region of CPS1 were used to identify two known and three previously unknown regions of high conservation, referred to enhancer/repressor regions A, B, and C. Highly conserved regions within the CPS1 5′ non-coding sequence include the proximal promoter, region A, the -enhancer, region B, and region C. (TIF) [file pone.0029527.s001.tif]

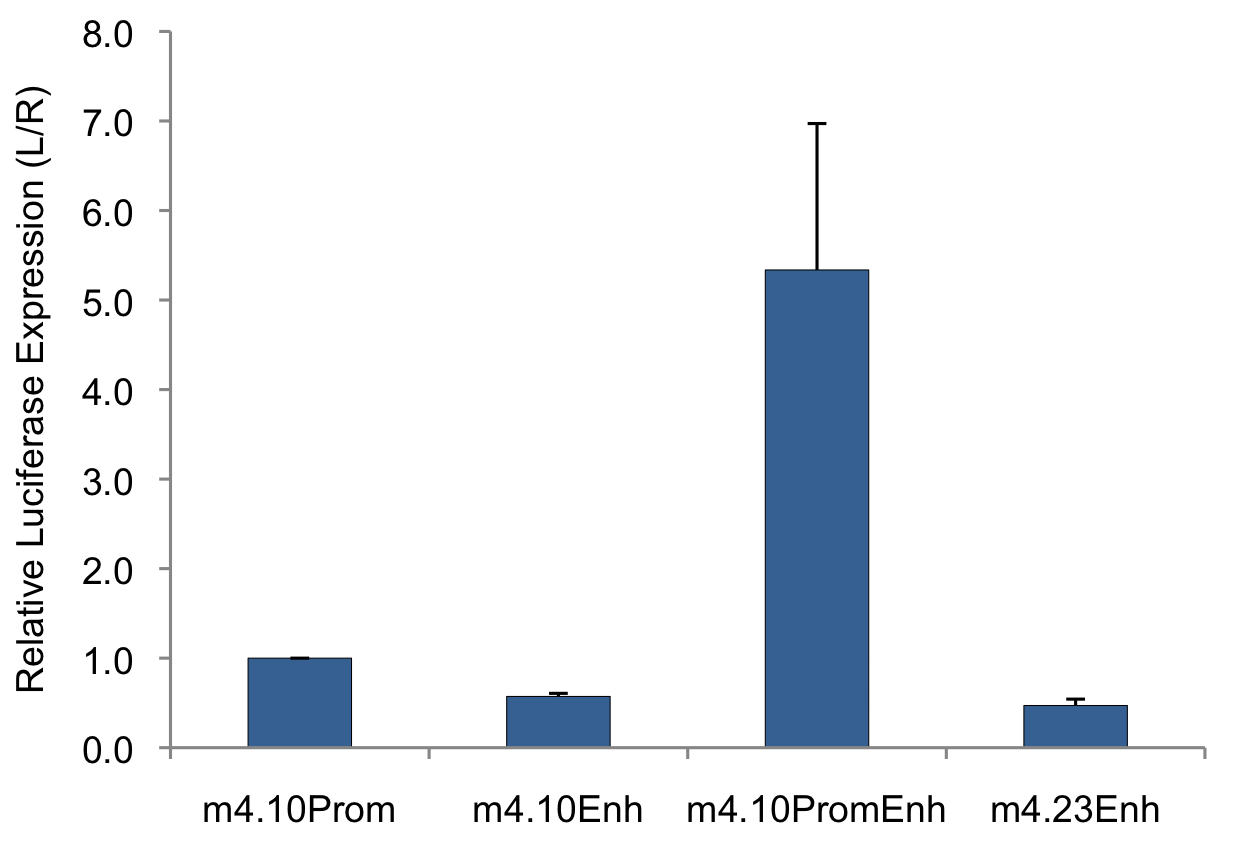

Supplement: Figure S2 — Highly conserved regulatory regions, upstream of the mouse Nags gene, function as promoter and enhancer elements. Mouse promoter (m4.10Prom), promoter and enhancer (m4.10PromEnh), and enhancer with TATA promoter (m4.23Enh) stimulated transcription while enhancer lacking a promoter (m4.10Enh) did not in liver cells. Calculated results are an average of three independent experiments that were carried out in triplicate, normalized to Rluc expression, and expressed relative to the promoter for each experiment with error reported as ±SEM. (TIF) [file pone.0029527.s002.tif]

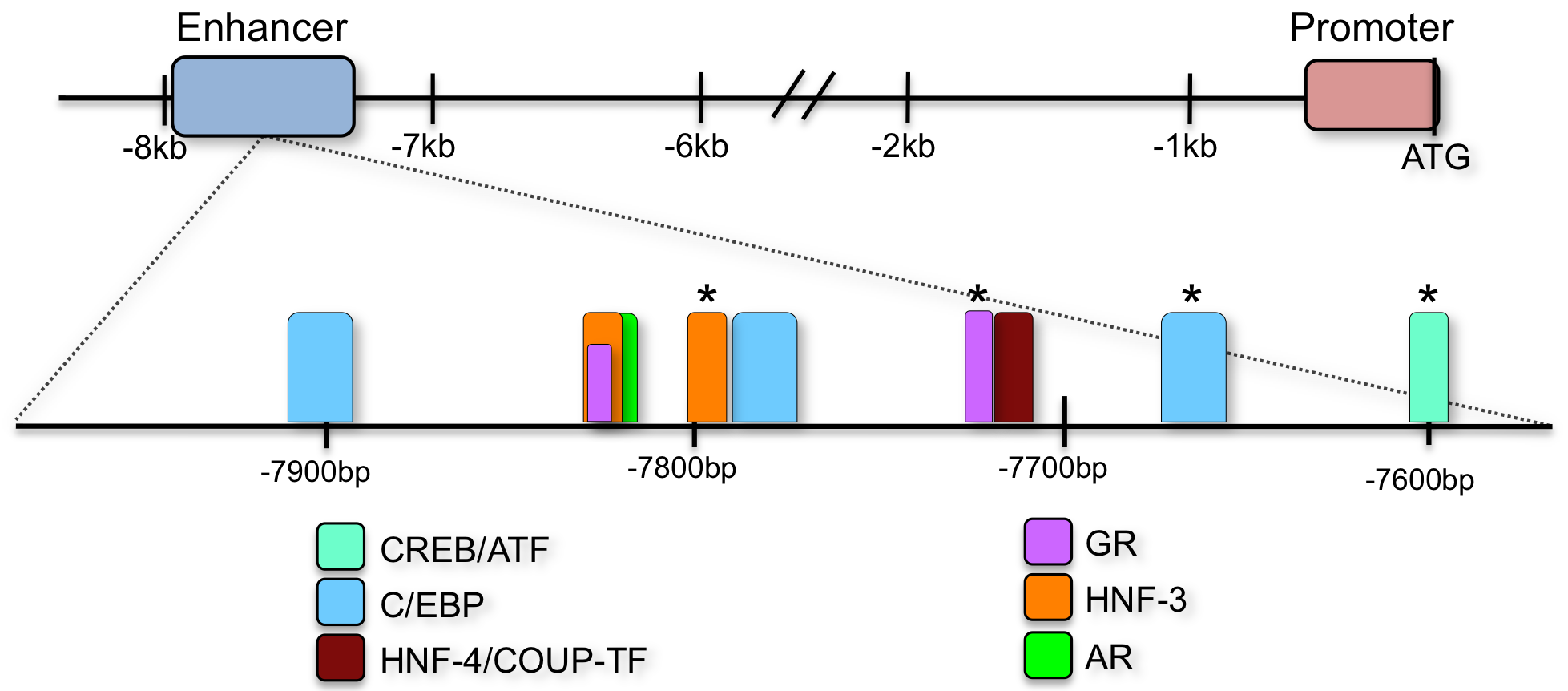

Supplement: Figure S3 — Novel transcription factor binding motifs, in the enhancer region of CPS1 , were identified using CLOVER. Several highly conserved transcription factor binding sites were present in the enhancer region. An asterisk denotes an experimentally verified transcription factor binding site. All motifs were spatially conserved between mammalian species. (TIF) [file pone.0029527.s003.tif]
