# Supplementary material for: Transcriptional Regulation of N-Acetylglutamate Synthase
Source: PLoS One. 2012 Feb 27;7(2):e29527. doi: 10.1371/journal.pone.0029527 (PMC3287996; doi:10.1371/journal.pone.0029527)
Supplement: Table S1 — Sequences of primers that were used to amplify human or mouse DNA by PCR for insertion of the promoter and enhancer regions into sequencing and reporter assay vectors. (DOCX) [file pone.0029527.s004.docx]

**Table S1.** Sequences of primers that were used to amplify human or mouse DNA by PCR for insertion of the promoter and enhancer regions into sequencing and reporter assay vectors.

| **Primer Name** | **Primer Sequence** |
| --- | --- |
| hPromXH Fw | 5’-CTCGAGTGGAGGCTGCAGTGAGCTATGATT-3’ |
| hPromXH Rv | 5’-AAGCTTTCGCCATGACGACAACCAACTCTT-3’ |
| hEnhXH Fw | 5’-CTCGAGAGGACCCTTCTGGGTGGAAGTTAT-3’ |
| hEnhXH Rv | 5’-AAGCTTTTCCTAGGGATCCACCCAATTCAGTC-3’ |
| hPromHXrev Fw | 5’-AAGCTTTGGAGGCTGCAGTGAGCTATGATT-3’ |
| hPromHXrev Rv | 5’-CTCGAGTCGCCATGACGACAACCAACTCTT-3’ |
| hEnhHXrev Fw | 5’-AAGCTTAGGACCCTTCTGGGTGGAAGTTAT-3’ |
| hEnhHXrev Rv | 5’-CTCGAGTTCCTAGGGATCCACCCAATTCAGTC-3’ |
| hEnhBS Fw | 5’-GGATCCAGGACCCTTCTGGGTGGAAGTTAT-3’ |
| hEnhBS Rv | 5’-GTCGACTTCCTAGGGATCCACCCAATTCAGTC-3’ |
| hCREBm Fw | 5’-GGGGGCAAGAGTTGGTCGACGTCATGGCGAAGC-3’ |
| hCREBm Rv | 5’-GCTTCGCCATGACGTCGACCAACTCTTGCCCCC-3’ |
| mPromXH Fw | 5’-CTCGAGGCTTAGGCTGGCCTTGAATTGCTT-3’ |
| mPromXH Rv | 5’-AAGCTTCCATGACGACAACCAAACCCACT-3’ |
| mEnhXH Fw | 5’-CTCGAGGGGAATGGCACTGAGACTGTGT-3’ |
| mEnhXH Rv | 5’-AAGCTTGCTCTCCCTTCCAAACATCTCTTCCT-3’ |
| mEnhBS Fw | 5’-GGATCCGGGAATGGCACTGAGACTGTGT-3’ |
| mEnhBS Rv | 5’-GTCGACGCTCTCCCTTCCAAACATCTCTTCCT-3’ |
