# Supplementary material for: Transcriptional Regulation of N-Acetylglutamate Synthase
Source: PLoS One. 2012 Feb 27;7(2):e29527. doi: 10.1371/journal.pone.0029527 (PMC3287996; doi:10.1371/journal.pone.0029527)
Supplement: Table S2 — Primer sequences used to determine transcription start sites of NAGS with 5′ RACE. Primers were designed according to manufacturer's instructions and used to determine transcription start sites of human and mouse NAGS in liver and small intestine RNA using 5′ RACE. (DOCX) [file pone.0029527.s005.docx]

**Table S2.** Primer sequences used to determine transcription start sites of *NAGS* with 5’ RACE. Primers were designed according to manufacturer’s instructions and used to determine transcription start sites of human and mouse *NAGS* in liver and small intestine RNA using 5’ RACE.

| **Primer Name** | **Primer Sequence** |
| --- | --- |
| hNAGS-GSP1 | 5’- GACGGCGAAGGGCTTGT-3’ |
| hNAGS-GSP2 | 5’- GTCTGGAACTGCGTGAGCC-3’ |
| hNAGS-GSP2B | 5’- CGACGCACTGGTTCAGGA-3’ |
| mNAGS-GSP1 | 5’- CATGACGGCGAAGGGCTTGT-3’ |
| mNAGS-GSP2 | 5’- GGTAGCAGGTCTGGAATTGCGTG-3’ |
| mNAGS-GSP2B | 5’- CGATGAGCCAGTGGCGTGCTTC-3’ |
