# Supplementary material for: Transcriptional Regulation of N-Acetylglutamate Synthase
Source: PLoS One. 2012 Feb 27;7(2):e29527. doi: 10.1371/journal.pone.0029527 (PMC3287996; doi:10.1371/journal.pone.0029527)
Supplement: Table S3 — Primer sequences used to generate DNA probes of the specified regions of m Nags . Primers were used to generate DNA probes, by PCR, of the promoter, enhancer, or non-specific specified regions of mNags. (DOCX) [file pone.0029527.s006.docx]

**Table S3.** Primer sequences used to generate DNA probes of the specified regions of m*Nags*. Primers were used to generate DNA probes, by PCR, of the promoter, enhancer, or non-specific specified regions of m*Nags*.

| **Primer Name** | **Primer Sequence** |
| --- | --- |
| mNAGS-Prom Region A Fw | 5’-AATCTGACCTTCGTGACCCTCACT-3’ |
| mNAGS-Prom Region A Rv | 5’- CACTCAGTCTCCGTGAGCCC-3’ |
| mNAGS-Prom Region B Fw | 5’-ACATTCCCAAATGTGGCCATCACC-3’ |
| mNAGS-Prom Region B Rv | 5’-AAAGCGCTCAGATGTCCTAGGTGT-3’ |
| mNAGS-Prom-NC Fw | 5’-AGCTGTACAGATGGTTGTGAGCCT-3’ |
| mNAGS-Prom-NC Rv | 5’-TCAGCGGGTAAGAGTACTGACTGCT-3’ |
| mNAGS-Enh Fw | 5’-GCTTTGTTGGAAGGTCAAGTCG-3’ |
| mNAGS-Enh Rv | 5’-GTGCCCTTCATCTTTGTCCCA-3’ |
| mNAGS-Enh-NC Fw | 5’-TAGGGCGTGTTCAAGACAGGGTTT-3’ |
| mNAGS-Enh-NC Rv | 5’- AGCTTGGTGGTGAGTGCCTTTAGT-3’ |
