# Supplementary material for: Transcriptional Regulation of N-Acetylglutamate Synthase
Source: PLoS One. 2012 Feb 27;7(2):e29527. doi: 10.1371/journal.pone.0029527 (PMC3287996; doi:10.1371/journal.pone.0029527)
Supplement: Table S4 — Primer sequences used for quantitative real-time PCR analysis of chromatin immunoprecipitation samples. (DOCX) [file pone.0029527.s007.docx]

**Table S4.** Primer sequences used for quantitative real-time PCR analysis of chromatin immunoprecipitation samples.

| **Primer Name** | **Primer Sequence** |
| --- | --- |
| mNAGS promoter FW | CATACACAAGGGGCGGAGA |
| mNAGS promoter RV | GGGTTCTTAACTTGCCGCTGA |
| mNAGS enhancer FW | GGCCTTCCGTAAGTAGGAAGCA |
| mNAGS enhancer RV | CCCACCTAGAGGGCTGTGT |
| MIP-2 FW | GAAGGGCAGGGCAGTAGAAT |
| MIP-2 RV | ATGCACGATGTCTGGAAAAG |
| mCPS1-CEBP Fw | GGAACATCTCTGGACATCA |
| mCPS1-CEBP Rv2 | CCAATTTGTTTGTAACCAGTGTGAA |
