# Supplementary material for: Transcriptional Regulation of N-Acetylglutamate Synthase
Source: PLoS One. 2012 Feb 27;7(2):e29527. doi: 10.1371/journal.pone.0029527 (PMC3287996; doi:10.1371/journal.pone.0029527)
Supplement: Table S5 — Results of CLOVER analysis of the enhancer region with sequence information for human and mouse CPS1 . Results were filtered to exclude motifs for transcription factors that are not expressed in the liver. (DOCX) [file pone.0029527.s008.docx]

**Table S5.** Results of CLOVER analysis of the enhancer region with sequence information for human and mouse *CPS1*. Results were filtered to exclude motifs for transcription factors that are not expressed in the liver.

| Sequence file: CPS1_enhancer_element.txt (5 sequences, 2875 bp, 40.4% C+G) | | | | |
| --- | --- | --- | --- | --- |
| Motif file: transfac_pro_n (588 motifs) | |  |  |  |
|  |  |  |  |  |
| Motif | Raw score | P-value from randomizing | |  |
| M00447\|AR | 10.8 | 0 |  |  |
| M00724\|HNF3alpha | 10.1 | 0 |  |  |
| M00912\|C/EBP | 6.34 | 0 |  |  |
| M00117\|C/EBPbeta | 6.07 | 0.001 |  |  |
| M00109\|C/EBPbeta | 5.83 | 0.002 |  |  |
| M00123\|c-Myc:Max | 4.52 | 0 |  |  |
| M00921\|GR | 4.09 | 0.002 |  |  |
| M00967\|HNF4, COUP | 3.64 | 0.004 |  |  |
| M00116\|C/EBPalpha | 3.44 | 0.006 |  |  |
| M00707\|TFIIA | 3.26 | 0.003 |  |  |
| M00981\|CREB, ATF | 2.52 | 0 |  |  |
| M00801\|CREB | 1.01 | 0 |  |  |
|  |  |  |  |  |
| Motif | Location | Strand | Sequence | Score |
| **>humanCPS1_enhancer** |  |  |  |  |
| M00921\|GR | 256 - 263 | + | cctgttct | 6.99 |
| M00981\|CREB, ATF | 264 - 272 | - | ctacgtcat | 8.05 |
| M00109\|C/EBPbeta | 325 - 338 | - | atgttgcaccacat | 7.08 |
| M00123\|c-Myc:Max | 331 - 342 | + | caccacatgctt | 7.87 |
| M00707\|TFIIA | 349 - 360 | - | gatcctcaaata | 6.31 |
| M00967\|HNF4, COUP | 375 - 383 | + | agggtccag | 7.47 |
| M00921\|GR | 385 - 392 | + | agtgtcct | 6.49 |
| M00109\|C/EBPbeta | 454 - 467 | - | atcttgcaaaatca | 7.41 |
| M00724\|HNF3alpha | 470 - 480 | + | tgtttactctt | 8.11 |
| M00724\|HNF3alpha | 489 - 499 | - | ttaagaaaaca | 6.11 |
| M00447\|AR | 508 - 522 | + | agagttgtttgttct | 6.57 |
| M00724\|HNF3alpha | 513 - 523 | + | tgtttgttctg | 9.29 |
| M00921\|GR | 515 - 522 | + | tttgttct | 6.65 |
| M00117\|C/EBPbeta | 560 - 573 | - | tcgttgtgcaaaga | 6.17 |
|  |  |  |  |  |
| **>mouseCPS1_enhancer** |  |  |  |  |
| M00109\|C/EBPbeta | 117 - 130 | + | agtttgtgaaagca | 6.03 |
| M00981\|CREB, ATF | 284 - 292 | - | caacgtcat | 6.98 |
| M00117\|C/EBPbeta | 311 - 324 | + | ctcttgcttcacta | 6.35 |
| M00967\|HNF4, COUP | 337 - 345 | + | caagtccat | 6.3 |
| M00109\|C/EBPbeta | 342 - 355 | - | ccattacacaacat | 8.34 |
| M00123\|c-Myc:Max | 372 - 383 | + | catcacacgtgt | 6.82 |
| M00967\|HNF4, COUP | 394 - 402 | + | agggtccag | 7.72 |
| M00921\|GR | 404 - 411 | + | agtgtcct | 6.49 |
| M00001\|MyoD | 428 - 439 | - | taccacctctct | 6.18 |
| M00189\|AP-2 | 440 - 451 | + | ctcctcatggcg | 6.06 |
| M00912\|C/EBP | 470 - 481 | + | cttgcaaaatca | 7.28 |
| M00724\|HNF3alpha | 486 - 496 | + | tgtttactctt | 7.9 |
| M00447\|AR | 522 - 536 | + | agagcagtttgttct | 11 |
| M00724\|HNF3alpha | 527 - 537 | + | agtttgttctg | 6.71 |
| M00921\|GR | 529 - 536 | + | tttgttct | 6.54 |
| M00921\|GR | 534 - 541 | + | tctgttca | 6.31 |
| M00707\|TFIIA | 548 - 559 | + | tataagaggggg | 8.67 |
| M00912\|C/EBP | 562 - 573 | - | ggttttggcaat | 6.11 |
