# Supplementary material for: Transcriptional Regulation of N-Acetylglutamate Synthase
Source: PLoS One. 2012 Feb 27;7(2):e29527. doi: 10.1371/journal.pone.0029527 (PMC3287996; doi:10.1371/journal.pone.0029527)
Supplement: Table S6 — Results of CLOVER analysis of the promoter region with sequence information for human and mouse NAGS . Results were filtered to exclude motifs for transcription factors that are not expressed in liver. (DOCX) [file pone.0029527.s009.docx]

**Table S6.** Results of CLOVER analysis of the promoter region with sequence information for human and mouse *NAGS*. Results were filtered to exclude motifs for transcription factors that are not expressed in liver.

| Sequence file: NAGS_promoter.txt (7 sequences, 7031 bp, 52.8% C+G) | | | |
| --- | --- | --- | --- |
| Motif file: transfac_pro_n (588 motifs) | |  |  |
|  |  |  |  |
| Motif | Raw score | P-value from randomizing | |
| M00933\|Sp1 | 20.3 | 0 |  |
| M00196\|Sp1 | 19.8 | 0 |  |
| M00932\|Sp1 | 19.7 | 0 |  |
| M00931\|Sp1 | 19.6 | 0 |  |
| M00255\|GC box | 14.7 | 0.0001 |  |
| M00257\|RREB-1 | 11.3 | 0.006 |  |
| M00008\|Sp1 | 8.38 | 0 |  |
| M00721\|CACCC-binding factor | 8.05 | 0.001 |  |
| M00691\|ATF1 | 5.05 | 0 |  |
| M00916\|CREB | 1.42 | 0.006 |  |
| M00017\|ATF | 1.03 | 0.003 |  |
|  |  |  |  |
| Motif | Location | Strand | Sequence |
| **>humanNAGS_promoter** |  |  |  |
| M00932\|Sp1 | 22 - 34 | + | atagggtgggact |
| M00932\|Sp1 | 138 - 150 | + | agtgggaggactg |
| M00008\|Sp1 | 233 - 242 | + | tgggcatggt |
| M00257\|RREB-1 | 244 - 257 | - | gtgtgcatttgtgg |
| M00255\|GC box | 302 - 315 | + | gggaggtggaggct |
| M00932\|Sp1 | 302 - 314 | + | gggaggtggaggc |
| M00257\|RREB-1 | 474 - 487 | - | aggggtgttttgag |
| M00916\|CREB | 542 - 555 | + | ggtaacctcatggt |
| M00255\|GC box | 607 - 620 | - | accacccgcccccg |
| M00932\|Sp1 | 608 - 620 | - | ccacccgcccccg |
| M00255\|GC box | 613 - 626 | - | cgcccccgccctcc |
| M00932\|Sp1 | 614 - 626 | - | gcccccgccctcc |
| M00932\|Sp1 | 618 - 630 | - | ccgccctcccact |
| M00935\|NF-AT | 646 - 655 | - | ctctttccag |
| M00932\|Sp1 | 850 - 862 | + | caggggcggggga |
| M00255\|GC box | 850 - 863 | + | caggggcgggggag |
| M00255\|GC box | 869 - 882 | - | tggccccgccccct |
| M00932\|Sp1 | 870 - 882 | - | ggccccgccccct |
| M00255\|GC box | 940 - 953 | - | ggaccccgccccga |
| M00932\|Sp1 | 941 - 953 | - | gaccccgccccga |
| M00255\|GC box | 961 - 974 | - | cagccccgcccaac |
| M00196\|Sp1 | 962 - 974 | - | agccccgcccaac |
| M01082\|BRCA1:USF2 | 1038 - 1045 | + | gttggttg |
| M00017\|ATF | 1041 - 1054 | - | ggttgtcgtcatgg |
| M00916\|CREB | 1042 - 1055 | + | gttgtcgtcatggc |
|  |  |  |  |
| **>mouseNAGS_promoter** |  |  |  |
| M00691\|ATF1 | 53 - 63 | - | tgagttcaagg |
| M00932\|Sp1 | 247 - 259 | - | atcaccgcccccc |
| M00932\|Sp1 | 252 - 264 | - | cgccccccccccc |
| M00257\|RREB-1 | 274 - 287 | - | gttttgttttgtgt |
| M01082\|BRCA1:USF2 | 566 - 573 | - | caacagga |
| M00255\|GC box | 598 - 611 | - | ggaccacaccccct |
| M00932\|Sp1 | 599 - 611 | - | gaccacaccccct |
| M00721\|CACCC-binding factor | 785 - 800 | - | ccatacacaaggggcg |
| M00932\|Sp1 | 793 - 805 | + | aaggggcggagaa |
| M00932\|Sp1 | 813 - 825 | - | ggcgccaccctct |
| M00257\|RREB-1 | 844 - 857 | + | cctcaaacgcaccc |
| M00255\|GC box | 882 - 895 | - | ccatcccgccccga |
| M00932\|Sp1 | 883 - 895 | - | catcccgccccga |
| M00721\|CACCC-binding factor | 959 - 974 | + | cgtcacctgtgggtgg |
| M00257\|RREB-1 | 965 - 978 | - | ctgtgggtgggggg |
| M00255\|GC box | 966 - 979 | + | tgtgggtggggggg |
| M00196\|Sp1 | 966 - 978 | + | tgtgggtgggggg |
| M00932\|Sp1 | 970 - 982 | + | ggtgggggggacg |
| M00932\|Sp1 | 974 - 986 | + | ggggggacgagtg |
| M00257\|RREB-1 | 982 - 995 | - | gagtgggtttggtt |
| M00257\|RREB-1 | 986 - 999 | - | gggtttggttgtcg |
| M01082\|BRCA1:USF2 | 989 - 996 | + | tttggttg |
| M00017\|ATF | 992 - 1005 | - | ggttgtcgtcatgg |
| M00916\|CREB | 993 - 1006 | + | gttgtcgtcatggc |
