# Supplementary material for: Transcriptional Regulation of N-Acetylglutamate Synthase
Source: PLoS One. 2012 Feb 27;7(2):e29527. doi: 10.1371/journal.pone.0029527 (PMC3287996; doi:10.1371/journal.pone.0029527)
Supplement: Table S7 — Results of CLOVER analysis of the enhancer region with sequence information for human and mouse NAGS. Results were filtered to exclude motifs for transcription factors that are not expressed in the liver. (DOCX) [file pone.0029527.s010.docx]

**Table S7.** Results of CLOVER analysis of the enhancer region with sequence information for human and mouse *NAGS*. Results were filtered to exclude motifs for transcription factors that are not expressed in the liver.

| Sequence file: NAGS_promoter.txt (7 sequences, 7031 bp, 52.8% C+G) | | | |
| --- | --- | --- | --- |
| Motif file: transfac_pro_n (588 motifs) | |  |  |
|  |  |  |  |
| Motif | Raw score | P-value from randomizing | |
| M00933\|Sp1 | 20.3 | 0 |  |
| M00196\|Sp1 | 19.8 | 0 |  |
| M00932\|Sp1 | 19.7 | 0 |  |
| M00931\|Sp1 | 19.6 | 0 |  |
| M00255\|GC box | 14.7 | 0.0001 |  |
| M00257\|RREB-1 | 11.3 | 0.006 |  |
| M00008\|Sp1 | 8.38 | 0 |  |
| M00721\|CACCC-binding factor | 8.05 | 0.001 |  |
| M00691\|ATF1 | 5.05 | 0 |  |
| M00117\|C/EBPbeta | 3.99 | 0.005 |  |
| M01082\|BRCA1:USF2 | 2.68 | 0.001 |  |
| M00916\|CREB | 1.42 | 0.006 |  |
| M00017\|ATF | 1.03 | 0.003 |  |
|  |  |  |  |
| Motif | Location | Strand | Sequence |
| **>humanNAGS_promoter** |  |  |  |
| M00932\|Sp1 | 22 - 34 | + | atagggtgggact |
| M00932\|Sp1 | 138 - 150 | + | agtgggaggactg |
| M00008\|Sp1 | 233 - 242 | + | tgggcatggt |
| M00257\|RREB-1 | 244 - 257 | - | gtgtgcatttgtgg |
| M00255\|GC box | 302 - 315 | + | gggaggtggaggct |
| M00932\|Sp1 | 302 - 314 | + | gggaggtggaggc |
| M00257\|RREB-1 | 474 - 487 | - | aggggtgttttgag |
| M00117\|C/EBPbeta | 526 - 539 | - | gagtttggcaaacc |
| M00916\|CREB | 542 - 555 | + | ggtaacctcatggt |
| M00255\|GC box | 607 - 620 | - | accacccgcccccg |
| M00932\|Sp1 | 608 - 620 | - | ccacccgcccccg |
| M00255\|GC box | 613 - 626 | - | cgcccccgccctcc |
| M00932\|Sp1 | 614 - 626 | - | gcccccgccctcc |
| M00932\|Sp1 | 618 - 630 | - | ccgccctcccact |
| M00935\|NF-AT | 646 - 655 | - | ctctttccag |
| M00932\|Sp1 | 850 - 862 | + | caggggcggggga |
| M00255\|GC box | 850 - 863 | + | caggggcgggggag |
| M00255\|GC box | 869 - 882 | - | tggccccgccccct |
| M00932\|Sp1 | 870 - 882 | - | ggccccgccccct |
| M00255\|GC box | 940 - 953 | - | ggaccccgccccga |
| M00932\|Sp1 | 941 - 953 | - | gaccccgccccga |
| M00255\|GC box | 961 - 974 | - | cagccccgcccaac |
| M00196\|Sp1 | 962 - 974 | - | agccccgcccaac |
| M01082\|BRCA1:USF2 | 1038 - 1045 | + | gttggttg |
| M00017\|ATF | 1041 - 1054 | - | ggttgtcgtcatgg |
| M00916\|CREB | 1042 - 1055 | + | gttgtcgtcatggc |
|  |  |  |  |
| **>mouseNAGS_promoter** |  |  |  |
| M00691\|ATF1 | 53 - 63 | - | tgagttcaagg |
| M00932\|Sp1 | 247 - 259 | - | atcaccgcccccc |
| M00932\|Sp1 | 252 - 264 | - | cgccccccccccc |
| M00257\|RREB-1 | 274 - 287 | - | gttttgttttgtgt |
| M01082\|BRCA1:USF2 | 566 - 573 | - | caacagga |
| M00255\|GC box | 598 - 611 | - | ggaccacaccccct |
| M00932\|Sp1 | 599 - 611 | - | gaccacaccccct |
| M00721\|CACCC-binding factor | 785 - 800 | - | ccatacacaaggggcg |
| M00932\|Sp1 | 793 - 805 | + | aaggggcggagaa |
| M00932\|Sp1 | 813 - 825 | - | ggcgccaccctct |
| M00257\|RREB-1 | 844 - 857 | + | cctcaaacgcaccc |
| M00255\|GC box | 882 - 895 | - | ccatcccgccccga |
| M00932\|Sp1 | 883 - 895 | - | catcccgccccga |
| M00721\|CACCC-binding factor | 959 - 974 | + | cgtcacctgtgggtgg |
| M00257\|RREB-1 | 965 - 978 | - | ctgtgggtgggggg |
| M00255\|GC box | 966 - 979 | + | tgtgggtggggggg |
| M00196\|Sp1 | 966 - 978 | + | tgtgggtgggggg |
| M00932\|Sp1 | 970 - 982 | + | ggtgggggggacg |
| M00932\|Sp1 | 974 - 986 | + | ggggggacgagtg |
| M00257\|RREB-1 | 982 - 995 | - | gagtgggtttggtt |
| M00257\|RREB-1 | 986 - 999 | - | gggtttggttgtcg |
| M01082\|BRCA1:USF2 | 989 - 996 | + | tttggttg |
| M00017\|ATF | 992 - 1005 | - | ggttgtcgtcatgg |
| M00916\|CREB | 993 - 1006 | + | gttgtcgtcatggc |
